# Estimated coverage of vaccines for children in Japan between 2011 and 2022: a descriptive study utilizing nationwide monthly market data

**DOI:** 10.1265/ehpm.25-00139

**Published:** 2025-10-09

**Authors:** Jun Miyata, Shingo Fukuma

**Affiliations:** 1 Department of Island and Community Medicine, Nagasaki University Graduate School of Biomedical Sciences, 205 Yoshikugicho, Goto, Nagasaki 853-8691, Japan; 2 Human Health Sciences, Kyoto University Graduate School of Medicine, 53 Shogoin-Kawaharacho, Sakyo, Kyoto, Kyoto 606-8507, Japan; 3 Department of Epidemiology, Infectious Disease Control and Prevention, Hiroshima University Graduate School of Biomedical and Health Sciences, 1-2-3 Kasumi, Minami, Hiroshima, Hiroshima 734-8553, Japan

**Keywords:** Drug utilization, Immunization program, Public financing, Vaccination coverage, Vaccination hesitancy, Voluntary program

## Abstract

**Background:**

Japan lacks comprehensive reports on the nationwide voluntary vaccine coverage. The effectiveness of public subsidies in promoting vaccination has not been fully investigated. Therefore, we aimed to estimate the nationwide coverage of voluntary vaccines, compare it with that of national immunization program (NIP)-included vaccines, and investigate the effectiveness of public subsidies.

**Methods:**

We obtained nationwide monthly vaccine market data for rotavirus, *Haemophilus influenzae* type b (Hib), diphtheria, tetanus toxoid, acellular pertussis, inactivated poliovirus (DTaP-IPV), and mumps vaccines; estimated recipient numbers; and calculated coverage as the proportion of children from October 2011 to March 2022. Regarding the NIP-included vaccine, we compared vaccine coverage calculated from nationwide annual market data with that estimated by World Health Organization (WHO)/United Nations Children’s Fund (UNICEF), using Bland-Altman analysis.

**Results:**

The estimates of Hib and DTaP-IPV vaccine coverage derived from market data were slightly higher than the WHO/UNICEF estimates, with mean differences of 0.05 (95% CI: 0.02–0.07) for Hib and 0.03 (95% CI: 0.01–0.05) for DTaP-IPV. The coverage of the rotavirus vaccine gradually increased long before the implementation of national subsidies, reaching 0.9 in 2020. Hib vaccine coverage had already achieved 1.0 by January 2012. The coverage of the DTaP-IPV vaccine was approximately 0.6–0.8 in 2013, reaching 1.0 in 2014. The coverage of mumps vaccine increased gradually from 2011 to 2021.

**Conclusions:**

Despite the possibility of overestimation, our estimates may serve as a valuable surrogate for actual vaccine coverage in Japan. An increasing trend in rotavirus and mumps vaccine coverage was observed when these vaccines were categorized as voluntary. Although vaccination policies differ from country to country, it would be beneficial to share findings on the impact of subsidies in Japan with other countries.

## Graphical Abstract

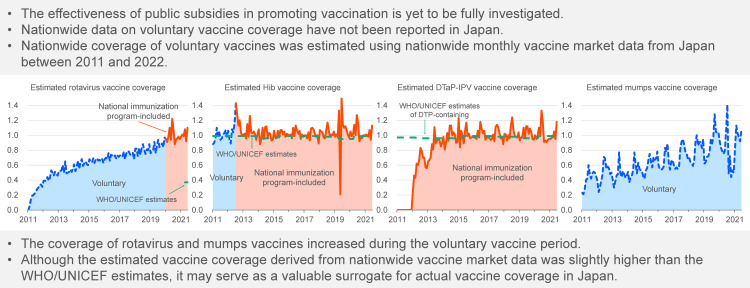

## Introduction

In 2019, the World Health Organization (WHO) identified vaccine hesitancy, characterized by delayed acceptance, reluctance, or refusal of vaccination despite the availability of services [1, 2], as one of the top ten threats to global health [1]. The determinants of vaccine hesitancy include costs, strength of recommendations, knowledge base, attitude of healthcare professionals, and design of vaccination programs [2].

The effectiveness of public subsidies, which may play a key role in enhancing vaccination coverage by reducing out-of-pocket expenses, incentivizing healthcare providers, and communicating the motivations of policymakers, has not been fully investigated. In Japan, approved vaccines are classified into two categories: those included in the national immunization program (NIP) and voluntary vaccines [3–5]. Under the Immunization Act, recipients of NIP-included vaccines incur no costs. For voluntary vaccines, some local governments use public funds to cover all or part of the vaccine fees; however, recipients are responsible for the out-of-pocket costs if local support is not provided. To evaluate the impact of public subsidies, it is essential to estimate and compare the nationwide coverage of voluntary vaccines with that of vaccines included in the NIP. However, while data on the nationwide coverage of NIP-included vaccines have been reported [6–8] under the Immunization Act, such data for voluntary vaccines are lacking. To address the lack of voluntary vaccine data, this study focused on nationwide monthly vaccine market data in Japan.

We estimated the nationwide coverage of voluntary vaccines, compared it with that of NIP-included vaccines, and investigated the effectiveness of public subsidies. We hypothesized that vaccine coverage of NIP-included vaccines would be higher than that of voluntary vaccines and that vaccine coverage would be increased by inclusion in the NIP. Although vaccination policies differ from country to country, it would be beneficial to share findings on the impact of subsidies in Japan with other countries.

## Methods

We obtained and examined the data on vaccines for children, including rotavirus, *Haemophilus influenzae* type b (Hib), diphtheria, tetanus toxoid, acellular pertussis, inactivated poliovirus (DTaP-IPV), and mumps vaccines, in Japan from October 2011 to March 2022. Focusing on vaccines for children allowed us to examine changes in vaccination coverage around the NIP implementation and to compare them with other vaccines for similar age groups. For rotavirus and Hib vaccines, we assessed the effectiveness of national subsidies by comparing vaccine coverage before and after their implementation. The rotavirus vaccine, categorized as voluntary since 2011, was incorporated into the NIP in October 2020 [3]. The Hib vaccine, categorized as voluntary since 2008, became part of the NIP in April 2013 [4]. The DTaP-IPV vaccine, which replaced DTaP and oral poliovirus vaccines, was introduced in November 2012 and was simultaneously included in the NIP [4]. The mumps vaccine was incorporated into the NIP-included measles, mumps, and rubella (MMR) vaccine from 1989 to 1993; however, the mumps component was subsequently removed, and a voluntary single mumps vaccine has been administered since 1993 [3] because of case reports of vaccine-related aseptic meningitis [9].

**Fig. 1:**
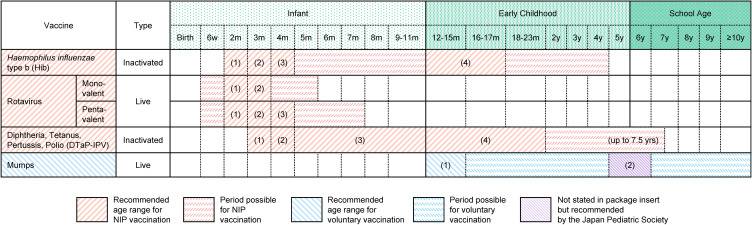
Excerpts from the vaccination schedule. The schedule was recommended by the Japan Pediatric Society, issued in October 2020 [3, 5]. Numbers in parentheses denote the doses of each vaccine.

Nationwide vaccination coverage among children was estimated as follows: 1. obtaining nationwide monthly vaccine market data; 2. estimating the number of vaccine recipients; and 3. calculating coverage as the proportion of recipients relative to the number of children newly eligible for vaccination, according to the definition used in Japan [6], cited by WHO/United Nations Children’s Fund (UNICEF) [7]. First, we utilized nationwide drug market records in the IQVIA IMSBase Japan Pharmaceutical Market (JPM) database, which includes data on drug names, forms, and country-level utilized doses collected from wholesalers purchasing and distributing drugs to medical facilities and pharmacies across Japan. Second, we estimated the number of vaccine recipients by dividing the utilized dose from the JPM database by the total dose typically administered to one child (four doses for the Hib and DTaP-IPV vaccines and two doses for the mumps vaccine) (Fig. 1) [3, 5]. For rotavirus vaccines, which have different dosing schedules for monovalent (Rotarix^®^) and pentavalent vaccines (RotaTeq^®^), the number of recipients was defined as the sum of estimates derived by dividing utilized doses by the total dose (two for the monovalent vaccine and three for the pentavalent vaccine) (Fig. 1). The annual number of recipients was calculated as the sum of monthly values from January through December. Third, we used the number of births from two months prior as a proxy for the number of children newly eligible for vaccination for rotavirus, Hib, and DTaP-IPV vaccines, and the number of births from one year prior as an indicator for the mumps vaccine, in accordance with recommended vaccination schedules (Fig. 1), to estimate vaccination coverage. Birth statistics were sourced from the Portal Site of Official Statistics of Japan [10]. The annual vaccination coverage was defined as the ratio of the total number of recipients to the total number of children newly eligible for vaccination in that year.

Regarding the NIP-included vaccine, we compared Hib coverage (2012–2021) calculated from nationwide annual market data with WHO/UNICEF estimates of Hib, and DTaP-IPV coverage (2015–2021) with WHO/UNICEF estimates of diphtheria, tetanus toxoid, and pertussis (DTP)-containing and poliovirus vaccines [8], employing Bland-Altman analysis to evaluate the differences and their 95% confidence intervals (CIs) [11].

In March and April 2020, at the onset of the coronavirus disease 2019 (COVID-19) pandemic, the number of administered NIP-included vaccines was lower than that in the same months in 2016–2019 in four cities in Japan [12]. Between April and autumn 2021, the supply of mumps vaccines was disrupted because of issues at the manufacturing plant of a pharmaceutical company [13].

Regarding the accuracy of the estimated vaccination coverage, mumps vaccine utilization data from the JPM database may include doses administered to adults because healthcare providers are recommended to receive two doses if they lack immunity unless there is documented proof of vaccination, serologic evidence of immunity, or laboratory confirmation of disease [14, 15]. Although a few reports from university hospitals have been published [16, 17], no nationwide statistics summarizing the number of vaccines administered to healthcare providers have been reported in Japan. Administration of Hib, DTaP-IPV, and rotavirus vaccines to adults is unlikely (Fig. 1) [3, 5]. Although Hib may be administered to adults with immunodeficiency [18], we considered the impact on the validity of the estimates to be limited.

All statistical tests were two-tailed, and the significance threshold was set at p < 0.05. All analyses were performed using R version 4.5.1 (R Foundation, Vienna, Austria).

The authors assert that all procedures contributing to this study comply with the ethical standards of the relevant national and institutional committees on human experimentation and the Helsinki Declaration of 1964 and its subsequent amendments. This study was exempt from ethical review and individual informed consent requirements because it involved a retrospective description of de-identified real-world data.

## Results

A comparison between the annual vaccine coverage calculated from nationwide market data and that estimated by the WHO/UNICEF is shown in Table 1. The estimates of Hib vaccine coverage derived from market data were slightly higher than the WHO/UNICEF estimates, with a mean difference of 0.05 (95% CI: 0.02–0.07). Similarly, market data-based estimates of DTaP-IPV coverage were higher than the WHO/UNICEF estimates of both DTP-containing vaccines (difference: 0.03, 95% CI: 0.01–0.05) and poliovirus vaccine (difference: 0.03, 95% CI: 0.01–0.05).

**Table 1:** Estimated annual vaccination coverage derived from market data and WHO/UNICEF estimates.

**Year**	**Estimates of vaccine coverage derived ** **from nationwide market data^a^**				**WHO/UNICEF estimates of vaccine coverage^b^**		
	
	**Rotavirus**	**Hib**	**DTaP-IPV^c^**	**Mumps**	**Hib**	**DTP-containing**	**Poliovirus**
2012	0.30	1.00	0.10	0.43	0.99	0.97	0.99
2013	0.48	1.11	0.67	0.50	0.99	0.96	0.99
2014	0.55	1.03	0.91	0.49	0.99	0.96	0.99
2015	0.63	1.01	1.02	0.54	0.96	0.96	0.99
2016	0.67	1.02	0.99	0.65	0.99	0.99	0.99
2017	0.72	1.02	1.01	0.65	0.99	0.99	0.97
2018	0.76	1.02	1.01	0.74	0.99	0.98	0.98
2019	0.81	1.02	1.02	0.80	0.98	0.98	0.98
2020	0.89	1.04	1.02	0.94	0.95	0.96	0.96
2021	1.00	1.03	1.01	0.80	0.99	0.99	0.99

DTaP, diphtheria, tetanus toxoid, and acellular pertussis; DTP, diphtheria, tetanus toxoid, and pertussis; Hib, *Haemophilus influenzae* type b; IPV, inactivated poliovirus; UNICEF, United Nations Children’s Fund; WHO, World Health Organization.

^a^Calculated as the proportion of children in the primary target demographic (using the number of births two months prior for rotavirus, Hib, and DTaP-IPV vaccines and the number of births one year prior for the mumps vaccine).

^b^Figures represent estimates of third-dose vaccine coverage sourced from WHO website [8]. Data on rotavirus and mumps vaccine coverage until 2021 are unavailable.

^c^The DTaP-IPV vaccine was implemented in November 2012, replacing DTaP and oral poliovirus vaccines.

**Fig. 2:**
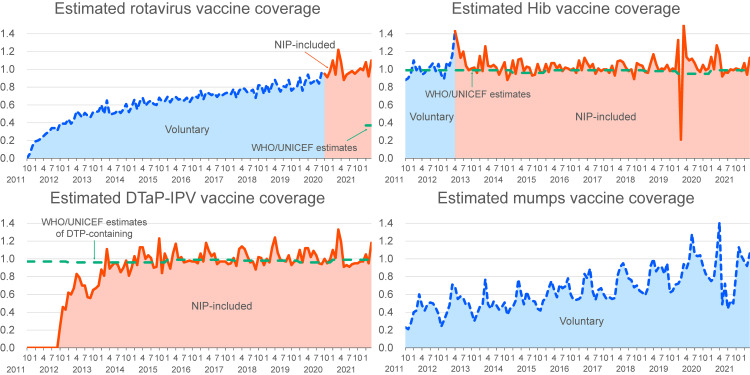
Estimated vaccination coverage trendline. The trendline was derived from nationwide monthly market data between 2012 and 2021 compared with World Health Organization (WHO)/United Nations Children’s Fund (UNICEF) estimates [8]. DTaP, diphtheria, tetanus toxoid, and acellular pertussis; DTP, diphtheria, tetanus toxoid, and pertussis; Hib, *Haemophilus influenzae* type b; IPV, inactivated poliovirus; NIP, national immunization program.

The estimated vaccination coverage during the study period is shown in Fig. 2 and Table 2. An upward trend in rotavirus vaccine coverage was evident long before the implementation of national subsidies, reaching 0.9 in January 2020 and stabilizing thereafter. Hib vaccine coverage, which became part of the NIP in April 2013, had already achieved 1.0 by the end of 2011. A temporary increase in coverage was observed with the implementation of the national subsidy, with coverage remaining around 1.0 subsequently. The estimated coverage of the DTaP-IPV vaccine was approximately 0.6–0.7 in 2013, reaching 1.0 in 2014 and then plateaued. Similar to the rotavirus vaccine, mumps vaccine coverage gradually increased from October 2011 to April 2021, when a short supply occurred, despite being consistently categorized as a voluntary vaccine.

**Table 2:** Estimated vaccination coverage derived from nationwide monthly market data between October 2011 and March 2022.

**Year**	**Month**	**Estimates of vaccination coverage^a^**			

		**Rotavirus**	**Hib**	**DTaP-IPV**	**Mumps**
2011	October	0.00	0.88	0.00	0.23
	November	0.05	0.90	0.00	0.21
	December	0.14	0.96	0.00	0.28
2012	January	0.19	1.10	0.00	0.40
	February	0.20	0.99	0.00	0.42
	March	0.22	1.04	0.00	0.60
	April	0.25	0.94	0.00	0.48
	May	0.28	0.96	0.00	0.42
	June	0.30	1.01	0.00	0.49
	July	0.34	1.03	0.00	0.51
	August	0.34	1.07	0.00	0.50
	September	0.31	0.98	0.00	0.45
	October	0.39	1.06	0.24	0.38
	November	0.39	0.94	0.46	0.24
	December	0.39	0.89	0.43	0.31
2013	January	0.44	1.08	0.62	0.39
	February	0.39	1.04	0.60	0.48
	March	0.43	1.16	0.67	0.71
	April	0.53	1.43	0.83	0.69
	May	0.50	1.29	0.79	0.55
	June	0.46	1.13	0.70	0.58
	July	0.51	1.20	0.70	0.56
	August	0.48	1.04	0.57	0.48
	September	0.46	0.99	0.56	0.50
	October	0.52	1.01	0.65	0.40
	November	0.52	1.02	0.67	0.30
	December	0.53	0.96	0.70	0.38
2014	January	0.60	1.15	0.88	0.46
	February	0.50	0.98	0.81	0.48
	March	0.65	1.26	1.11	0.77
	April	0.49	1.04	0.89	0.49
	May	0.50	1.03	0.93	0.44
	June	0.51	1.05	0.96	0.52
	July	0.54	1.02	0.93	0.48
	August	0.51	0.94	0.85	0.43
	September	0.55	1.01	0.90	0.46
	October	0.61	1.08	0.98	0.51
	November	0.52	0.88	0.81	0.37
	December	0.58	0.95	0.90	0.44
2015	January	0.66	1.10	1.03	0.46
	February	0.55	0.95	0.93	0.53
	March	0.64	1.11	1.13	0.77
	April	0.68	1.12	1.13	0.61
	May	0.59	0.98	1.00	0.49
	June	0.63	1.03	1.04	0.63
	July	0.65	1.02	1.01	0.55
	August	0.61	0.93	0.90	0.53
	September	0.60	0.94	0.91	0.52
	October	0.68	1.06	1.23	0.44
	November	0.64	0.97	0.84	0.42
	December	0.64	0.96	1.06	0.53
2016	January	0.69	1.06	0.98	0.56
	February	0.63	0.97	0.89	0.67
	March	0.68	1.07	1.06	0.75
	April	0.69	1.07	1.17	0.65
	May	0.66	1.01	0.99	0.57
	June	0.67	1.05	1.02	0.71
	July	0.65	0.98	0.96	0.67
	August	0.68	1.02	0.97	0.69
	September	0.63	1.01	0.98	0.78
	October	0.68	0.99	0.97	0.60
	November	0.70	1.01	0.97	0.54
	December	0.68	0.96	0.95	0.54
2017	January	0.76	1.10	1.06	0.55
	February	0.66	0.99	0.97	0.58
	March	0.74	1.13	1.18	0.83
	April	0.73	1.07	1.09	0.78
	May	0.71	1.02	1.03	0.89
	June	0.73	1.06	1.06	0.63
	July	0.69	0.95	0.94	0.54
	August	0.73	1.01	0.97	0.65
	September	0.71	0.98	0.97	0.67
	October	0.73	1.00	0.97	0.60
	November	0.74	1.00	0.94	0.55
	December	0.72	0.96	0.95	0.57
2018	January	0.78	1.04	1.01	0.55
	February	0.68	0.93	0.92	0.56
	March	0.76	1.09	1.11	0.81
	April	0.79	1.10	1.14	0.91
	May	0.78	1.08	1.11	0.95
	June	0.74	1.02	1.03	0.88
	July	0.75	0.98	0.96	0.78
	August	0.77	1.00	0.97	0.76
	September	0.68	0.89	0.88	0.68
	October	0.82	1.07	1.02	0.70
	November	0.82	1.05	1.01	0.64
	December	0.72	1.04	0.93	0.62
2019	January	0.83	0.97	1.00	0.59
	February	0.74	0.96	0.96	0.67
	March	0.83	1.07	1.13	0.92
	April	0.88	1.17	1.24	1.00
	May	0.81	1.06	1.07	0.86
	June	0.75	0.98	0.99	0.91
	July	0.81	1.01	0.98	0.91
	August	0.80	1.00	0.97	0.83
	September	0.88	1.05	1.04	0.95
	October	0.76	0.98	0.93	0.62
	November	0.79	1.01	0.97	0.64
	December	0.80	0.99	0.98	0.71
2020	January	0.91	1.33	1.10	0.72
	February	0.76	0.21	0.96	0.77
	March	0.86	1.50	1.12	0.94
	April	0.94	1.14	1.12	0.89
	May	0.84	1.10	1.02	0.98
	June	0.86	1.12	1.09	1.28
	July	0.89	1.07	1.04	1.05
	August	0.84	1.00	0.95	1.03
	September	0.97	0.97	0.94	1.04
	October	0.95	1.05	1.00	0.92
	November	0.91	0.99	0.95	0.83
	December	0.98	1.01	0.99	0.79
2021	January	1.10	1.14	1.10	0.75
	February	0.94	1.00	1.01	0.79
	March	1.22	1.27	1.33	1.06
	April	1.08	1.17	1.19	1.41
	May	0.88	0.92	0.91	0.49
	June	0.94	0.97	0.93	0.72
	July	0.96	0.96	0.91	0.44
	August	0.98	1.00	0.94	0.53
	September	0.95	0.99	0.95	0.50
	October	0.98	1.01	0.95	0.82
	November	1.01	1.00	0.97	1.13
	December	0.99	0.98	0.97	1.03
2022	January	1.08	1.07	1.05	0.97
	February	0.92	0.94	0.95	0.92
	March	1.10	1.13	1.18	1.06

DTaP, diphtheria, tetanus toxoid, and acellular pertussis; Hib, *Haemophilus influenzae* type b; IPV, inactivated poliovirus.

^a^Calculated as the proportion of children in the primary target demographic (using the number of births two months prior for rotavirus, Hib, and DTaP-IPV vaccines and the number of births one year prior for the mumps vaccine).

## Discussion

This study highlights two important findings. Estimates of vaccine coverage derived from market data were slightly higher than the corresponding WHO/UNICEF estimates. The estimated coverage of rotavirus and mumps vaccines increased steadily from October 2011 to the end of 2020, despite their categorization as voluntary vaccines.

For evidence-based vaccination policies, it is important to estimate nationwide coverage of voluntary vaccines. Although the estimated vaccine coverage derived from nationwide vaccine market data was slightly higher than the WHO/UNICEF estimates (Table 1), it may serve as a valuable surrogate for actual vaccine coverage in Japan, particularly for voluntary vaccines, for which national statistical data are limited. Local governments may possess coverage data only for specific regions (not nationwide) or may lack data altogether; meanwhile, it is difficult to collect nationwide data from medical institutions and recipients. The higher estimates could be accounted for, in part, by vaccines discarded by medical institutions due to expiration or other reasons, as well as doses administered to older children or adults for catch-up vaccination [3, 14–17] or for immunodeficiency [18]. To estimate vaccination coverage from market data more effectively, it is necessary to obtain comprehensive data on vaccine waste and the vaccination of healthcare workers. Another limitation of the nationwide monthly vaccine market data is that they may not correctly reflect the timing of vaccinations owing to potential delays between marketing and actual vaccination.

An upward trend in the coverage of rotavirus and mumps vaccines was exhibited during the period when they were categorized as voluntary, inconsistent with our hypothesis that vaccine coverage would be increased by inclusion in the NIP. Several factors might have contributed to this increase: 1. increase in local government public subsidies; 2. proactive vaccine communication by healthcare providers; and 3. increased public awareness among caregivers of vaccine recipients (children) and other stakeholders. The DTaP-IPV vaccine, which was included in the NIP from the outset, showed a sharp increase in estimated coverage after its introduction in November 2012, and the estimates have remained close to 1.0 since April 2014, when the preceding DTaP vaccine was discontinued. The effectiveness of local subsidies was observed in a prior study using publicly available data from Nagoya City in Japan, which indicated that mumps vaccine coverage in one-year-old children increased from 24% in fiscal year 2010, when local subsidies began, to 91% in fiscal year 2016 [19]. Besides economic factors, factors that encourage healthcare providers, children, and their caregivers are also important. A nationwide cross-sectional study based on a web-based, self-administered questionnaire in Japan revealed that primary care physicians who worked in areas with mumps vaccine subsidies were 2.4 times more likely to recommend the mumps vaccine to children than those in areas without subsidies [20]. A cross-sectional study in Japan that interviewed parents during 18-month health checkups for children revealed that pediatricians’ recommendations and local government newsletters on vaccines were positively associated with rotavirus vaccination [21].

Although no significant changes in rotavirus or Hib vaccine coverage were observed at the implementation of national subsidies (October 2020 for rotavirus and April 2013 for Hib), national subsidies are believed to be effective. A cross-sectional study in Japan reported a positive association between household income and voluntary vaccination uptake among children in a city without subsidies but not where subsidies were available [22]. Other studies have indicated a positive association between household income and vaccination in Japan [21, 23] and other countries [24, 25]. To mitigate these inequities, evidence-based vaccines should be included in the NIP as early as possible.

Categorization of vaccines can lead to differences in vaccination coverage. In France, vaccines implemented before 1966 (diphtheria, tetanus, and poliovirus vaccines) were categorized as mandatory vaccines, and later vaccines, such as measles, mumps, and rubella vaccines, were categorized as recommended vaccines because of their historical background until 2018, leading to lower coverage of the latter vaccines, even though both vaccines were subsidized by the government and recipients did not have to pay for them [26]. After several vaccines previously categorized as recommended became mandatory in 2018 [26, 27], an increase in the coverage of these vaccines was observed [6, 8, 27]. In Japan, it may have the effect of announcing to the public that voluntary vaccines have a lower priority to separate NIP-included and voluntary vaccines.

In conclusion, we estimated nationwide coverage of voluntary vaccines using nationwide monthly drug market data from Japan between 2011 and 2022. Because the estimated values were slightly higher than those reported by WHO/UNICEF, estimates of vaccine recipients and coverage derived from market data may be subject to overestimation. An increasing trend in the estimated coverage of rotavirus and mumps vaccines was observed from October 2011 to the end of 2020, despite these vaccines being categorized as voluntary. No significant changes in Hib vaccine coverage were observed with the implementation of national subsidies; however, such subsidies might still play a role. The coverage of the DTaP-IPV vaccine, which was included in the NIP from the outset, showed a sharp increase following its introduction, suggesting the effectiveness of national subsidy. The current findings on the estimated vaccination coverage trend, particularly the observed increase in coverage of rotavirus and mumps vaccines during the period when they were categorized as voluntary, will be beneficial in different situations in other countries. To address these inequities, it is important to include effective vaccines in the NIP as soon as possible.

## Abbreviations

CI: confidence interval
COVID-19: coronavirus disease 2019
DTaP: diphtheria, tetanus toxoid, and acellular pertussis
DTP: diphtheria, tetanus toxoid, and pertussis
Hib: *Haemophilus influenzae* type b
IPV: inactivated poliovirus
JPM: Japan Pharmaceutical Market
MMR: measles, mumps, and rubella
NIP: national immunization program
UNICEF: United Nations Children’s Fund
WHO: World Health Organization

## Declarations

### Availability of data and material

Raw nationwide drug market data from the IQVIA IMSBase JPM database are not available from the corresponding author owing to commercial restrictions. To access these data, please visit the IQVIA website (https://www.iqvia.com/).
